# Chronic colitis upregulates microRNAs suppressing brain-derived neurotrophic factor in the adult heart

**DOI:** 10.1371/journal.pone.0257280

**Published:** 2021-09-20

**Authors:** Yanbo Tang, Kevin T. Kline, Xiaoying S. Zhong, Ying Xiao, Haifeng Lian, Jun Peng, Xiaowei Liu, Don W. Powell, Guodu Tang, Qingjie Li

**Affiliations:** 1 Department of Gastroenterology, the First Affiliated Hospital, Guangxi Medical University, Nanning, China; 2 Division of Gastroenterology, Department of Internal Medicine, The University of Texas Medical Branch at Galveston, Galveston, TX, United States of America; 3 Department of Gastroenterology, Xiangya Hospital, Central South University, Changsha, China; 4 Department of Gastroenterology, Binzhou Medical University Hospital, Binzhou, China; 5 Department of Pharmacology, Xiangya School of Pharmaceutical Sciences, Central South University, Changsha, China; Future University, EGYPT

## Abstract

Ulcerative colitis and Crohn’s disease are classified as chronic inflammatory bowel diseases (IBD) with known extraintestinal manifestations. The interplay between heart and gut in IBD has previously been noted, but the mechanisms remain elusive. Our objective was to identify microRNAs mediating molecular remodeling and resulting cardiac impairment in a rat model of colitis. To induce chronic colitis, dextran sodium sulfate (DSS) was given to adult rats for 5 days followed by 9 days with normal drinking water for 4 cycles over 8 weeks. Echocardiography was performed to evaluate heart function. DSS-induced colitis led to a significant decrease in ejection fraction, increased left ventricular mass and size, and elevated B-type natriuretic protein. MicroRNA profiling showed a total of 56 miRNAs significantly increased in the heart by colitis, 8 of which are predicted to target brain-derived neurotrophic factor (BDNF). RT-qPCR validated the increases of miR-1b, Let-7d, and miR-155. Transient transfection revealed that miR-155 significantly suppresses BDNF in H9c2 cells. Importantly, DSS colitis markedly decreased BDNF in both myocardium and serum. Levels of various proteins critical to cardiac homeostasis were also altered. Functional studies showed that BDNF increases cell viability and mitigates H_2_O_2_-induced oxidative damage in H9c2 cells, demonstrating its protective role in the adult heart. Mechanistically, cellular experiments identified IL-1β as the inflammatory mediator upregulating cardiac miR-155; this effect was confirmed in adult rats. Furthermore, IL-1β neutralizing antibody ameliorated the DSS-induced increase in miR-155 and concurrent decrease in BDNF in the adult heart, showing therapeutic potential. Our findings indicate that chronic colitis impairs heart function through an IL-1β→miR-155→BDNF signaling axis.

## Introduction

Inflammatory bowel disease (IBD) represents a spectrum of chronic inflammatory mediated conditions, including Crohn’s disease and ulcerative colitis, manifesting in varying degrees and locations of colitis. While therapeutic research has been primarily targeted on affecting the pathways yielding abnormal colonic inflammation, IBD has known extraintestinal manifestations mediated through a chronic inflammatory and hypercoagulable state [1]. Chronic inflammation has previously been identified as atherogenic with increased risk of developing cardiovascular disease. The interplay between patients with heart failure (HF) and consequential alterations in colonic integrity and mucosal bacteria burden has also been noted [2]. In a Danish cohort study, patients with IBD had an increased risk of hospitalization for HF, a risk strongly correlated to periods of active disease [3]. Patients with IBD diagnosed for greater than a year were noted to have an increased incidence of coronary artery disease events compared to controls despite having fewer risk factors for coronary artery disease [4]. In addition, Crohn’s disease and ulcerative colitis have been identified as independent risk factors for acute myocardial infarction [5]. Despite the independent recognition of IBD as a risk factor for heart disease, the mechanisms mediating the changes in cardiac function in patients with IBD have not been well elucidated, partly due to unavailability of human heart tissue.

Cardiac remodeling in the presence of chronic inflammation has been investigated as an important pathway eventually mediating physiologic HF. TNF-α, an important inflammatory cytokine in the pathogenesis of IBD and a well-established therapeutic target for both Crohn’s disease and ulcerative colitis, has been linked to left ventricle remodeling [6]. While anti-TNF-α therapy is effective in reducing disease activity and inducing clinical remission in IBD patients, adverse events including heart failure have been observed [7,8]. This suggests that TNF-α signaling has a complex role in cardiac homeostasis and that other proinflammatory mediators such as interleukin 1beta (IL-1β) or lipopolysaccharide (LPS), which are increased in IBD, could play a role in molecular remodeling in the adult heart. Clarifying how these inflammatory mediators induce cardiac remodeling may lead to development of novel therapeutic strategies.

MiRNAs have been related to posttranscriptional regulation of gene expression in major cardiac physiological and pathological processes, and cardiac muscle phenotypes are tightly regulated by miRNAs to maintain cardiac homeostasis [9]. miR-155 plays a key role in the homeostasis and function of the immune system [10], but abnormal high levels of circulating miR-155 found in patients with ulcerative colitis could be detrimental [11]. While miR-155 knockout mice demonstrated resistance to developing cardiac hypertrophy [12], cardiac miRNA profiles in the presence or absence of chronic colitis have not been characterized. Whether and how miR-155 mediates cardiac remodeling in IBD remain to be determined.

Brain-derived neurotrophic factor (BDNF) has been identified as a neurotrophin mediating wide-ranging effects on the cardiovascular system including cardiac and coronary artery development and maintenance of homeostasis including protection against reactive oxygen species [13]. In adult mammals, BDNF governs autonomic transmission to the heart and exerts prominent angiogenic effects [14]. Of note, the BDNF receptor, tropomyosin-related kinase receptor B (TrkB), exists in the myocardium [15]. BDNF/TrkB signaling is required for the heart to fully contract and relax [14,16]. Recent studies suggest that alterations in BDNF synthesis and function are associated with increased incidence of heart failure, arrhythmia, and myocardial infarction, indicating its necessity in the cardiovascular system [17–19]. It is unclear, however, if chronic colitis suppresses BDNF by upregulating certain miRNAs. The aim of this study was to determine whether and how colitis alters miRNA profiles in the adult heart, and if changes in miRNAs are associated with alterations in functional proteins. Our results indicate that chronic colitis upregulates IL-1β augmenting certain cardiac miRNAs, particularly miR-155, which leads to suppression of BDNF corresponding to impaired heart function. Our findings reinforce the clinically noted connection of IBD and HF and suggest that cardiac impairment in IBD could be mitigated with IL-1β antibody.

## Materials and methods

### Reagents

Dextran sulfate sodium (DSS), molecular weight 36-50K, was purchased from Gojira Fine Chemicals (Bedford Heights, OH). LPS, H_2_O_2_, and 2,4,6-trinitrobenzene sulfonic acid (TNBS) were purchased from Sigma (St. Louis, MO). Rat recombinant IL-1β (Cat. # Z03014) and TNF-α (Cat. # Z02999) were purchased from GenScript (Piscataway, NJ) and neutralizing IL-1β antibody (Cat. # AF-401-NA) was from R&D Systems (Minneapolis, MN).

### Cell culture

H9c2 rat cardiaomyoblast cells were purchased from American type culture collection (ATCC, Manassas, VA) and maintained in Dulbecco’s modified eagle medium supplemented with 10% fetal bovine serum and 0.1% penicillin-streptomycin solution. Transient transfection was performed using Lipofectamine RNAiMAX (Thermo Fisher Scientific, Waltham, MA) [20].

### Animals

Total 70 6-week old male Sprague Dawley rat littermates (150 to 200 grams) were purchased from Harlan (Indianapolis, IN) and used in the preclinical studies. The rats were housed in the UTMB animal facility with 12h light/12 dark cycle, a temperature range of 24–26°C, and a relative humidity of 40–70%. Regular chow and water were provided *ad libitum*.

#### Protocols for animal experiments

Three colitis models were used in this project. 1. Chronic colitis (n = 8 per group). Chronic colitis was induced with 3% DSS, which was applied for 4 cycles of 5 days each, with intermittent 9-day intervals of normal drinking water. 2. Acute TNBS colitis (n = 6 per group). Rats were lightly anesthetized with isoflurane (4% for induction and 1% for maintenance) and 250 μl of TNBS in phosphate buffered saline containing 40% ethanol was injected intrarectally via a catheter, advanced to 8 cm into the colon [21,22]. Control rats received one time intracolonical injection of 250 μl of saline. 3. Acute DSS colitis (n = 6 per group). To determine if IL-1β antibody ameliorates DSS-induced miR-155 upregulation, 6-week old male Sprague Dawley rats were treated with 5% DSS in the drinking water for 7 days. IL-1β antibody (25 μg/kg in 200 μl of saline) was administrated daily by intraperitoneal injection for 7 days. Rats in control groups were subjected to daily intraperitoneal injection of 200 μl saline. Animals were euthanized 3 hours after the last dose of IL-1β antibody. The individual hearts were collected, snap-frozen in liquid nitrogen, and pulverized for RNA extraction. All animals with experimental colitis were monitored daily by a member of the research team for assessment of food consumption and any unexpected discomfort or morbidity. At the end of each experiment, animals were decapitated under deep plane of anesthesia with 4% isoflurane.

To assess the impact of IL-1β and TNF-α on the miR-155 levels in the adult heart, 6-week old male Sprague Dawley rats were anesthetized with isoflurane (4% for induction and 1% for maintenance) and given recombinant IL-1β or TNF-α (GeneScript, 10 μg/kg) by tail vein injection. Control group received saline (n = 8 per group). Twenty-four hours later, animals were decapitated under deep plane of anesthesia with 4% isoflurane. Individual hearts were removed and snap-frozen in liquid nitrogen, followed by pulverization with mortar and pestle. A small aliquot of the pulverized cardiac tissue was used for isolation of total RNA containing miRNA.

#### Ethics statement

This study was carried out in strict accordance with the recommendations in the Guide for the Care and Use of Laboratory Animals of the National Institutes of Health. All procedures were approved by the Institutional Animal Care and Use Committee, The University of Texas Medical Branch at Galveston (Protocol # 1512071A).

### Rat echocardiography

Rat cardiac function was examined via trans-thoracic echocardiography using a Vevo 770 High-Resolution Imaging System equipped with a 30 mHz probe (VisualSonics, Toronto, Canada). The rats were anesthetized with isoflurane (4% for induction and 1% for maintenance) and positioned in left lateral decubitus. The anterior chest hair was removed with Nair Hair Remover Lotion (Church & Dwight Co., Ewing, NJ). ECG was monitored throughout the experiment and temperature was kept at 37°C on the heated plate. The left ventricular short-axis and long-axis view and the apical four-chamber view were examined by two-dimensional and M-mode echocardiography. A number of cardiac function-related parameters were determined, including left ventricular ejection fraction (LVEF), LV mass, and LV size (short-axis area and long-axis length).

### Immunohistochemistry

For histologic examination, a full-thickness colon specimen and half of the heart were obtained, fixed in 10% formalin, embedded in paraffin, sectioned, and de-waxed prior to histochemical staining stained with hematoxylin and eosin (H&E) [23]. For immunohistochemistry, five-micrometer sections were baked in a 50–55°C oven for 1 hour. After antigen retrieval and one-hour blocking with 10% serum, the slides were treated with anti-COL3A1 mouse monoclonal antibody (sc-514601, Santa Cruz Biotechnology; 1:50 dilution) overnight, followed by washing three times 15 min each with 1X PBS. The sections were then incubated for 1 hour at room temperature with ALEXA-conjugated antibody (Invitrogen) diluted 1:400 in PBS. Images were captured using a LEICA DMI 6000 B microscope. Terminal deoxynucleotidyl transferase (TdT) dUTP Nick-End Labeling (TUNEL) assay was performed using the TUNEL Assay Kit—HRP-DAB (ab206386, abcam, Cambridge, UK) and following the protocol provided by the manufacturer.

### Real-time reverse transcriptase-polymerase chain reaction (RT-qPCR)

Total RNA including miRNA was extracted using the miRNeasy Mini Kit (QIAGEN, Valencia, CA), followed by cDNA synthesis using SuperScript III First-Strand Synthesis System or TaqMan™ Advanced miRNA cDNA Synthesis Kit (Thermo Fisher Scientific, Waltham, MA) [23]. The mRNA levels were quantitated using SYBR Green-based qPCR with *Gapdh* as internal control and miRNA levels determined using TaqMan-based qPCR with 18S ribosomal RNA as internal control. Primers are as follows: *Bdnf*-F, 5’-TAC CTG GAT GCC GCA AAC AT-3’, *Bdnf*-R, 5’-GCT GTG ACC CAC TCG CTA AT-3’; *Il1b*-F, 5’-CAG GAT GAG GAC CCA AGC AC-3’, *Il1b*-R, 5’-GTC GTC ATC ATC CCA CGA GT-3’; *Mmp7*-F, 5’-CTC TCT GGG TCT GGG TCA CT-3’, *Mmp7*-R, 5’-AAG GGC GTT TGC TCA TTC CA-3’; *Mmp9*-F, 5’-CTG GGC ATT AGG GAC AGA GGA-3’, *Mmp9*-R, 5’-GAC ACT GAG AAT CCC TGA GCG-3’; *Il18*-F, 5’-CAA AAG AAA CCC GCC TGT GTT-3’, *Il18*-R, 5’-AGT CTG GTC TGG GAT TCG TTG-3’.

### Myeloperoxidase (MPO) assay

Frozen colon mucosa/submucosa was pulverized in liquid nitrogen, homogenized in 20 mM phosphate buffer (pH 7.4), and centrifuged at 4°C for 10 minutes. Pellets were sonicated in 50 mM phosphate buffer (pH 6.0) containing 0.5% hexadecyl trimethyl ammonium bromide and centrifuged at 4°C for 5 minutes. The supernatant (100 μl) was incubated with 16 mM tetramethyl benzidine in 50% ethanol, 0.3 mM H_2_O_2_, and 8 mM sodium phosphate buffer (pH 5.4) for 3 minutes. Myeloperoxidase activity was measured by reading the absorbance at 655 nm in a microplate reader [22].

### Subcellular fractionation

Frozen tissue was smashed into fine powder in liquid nitrogen. Around 100 mg of pulverized tissue was transferred to a microcentrifuge tube and about 10 times tissue weight of ice-cold cytoplasmic extract buffer (250 mM Sucrose, 20 mM HEPES, 10 mM KCl, 1.5 mM MgCl_2,_ 1 mM EDTA, 1 mM EGTA, 1 mM DTT) containing protease inhibitors was added to each tube. The tissue was homogenized for 20 seconds with a Polytron homogenizer. After incubation on ice for 20 min, the nuclear pellet was centrifuged out at 720 g (3000 rpm) for 5 min. The nuclear pellet was washed once with the cytoplasmic extract buffer and resuspended with the nuclear extract buffer (20 mM Tris HCl, 420 mM NaCl, 1.5 mM MgCl_2,_ 0.2 mM EDTA, 10% glycerol, 0.1% SDS, 1 mM PMSF). The nuclei was incubated on ice for 40 min and vortexed on the highest setting for 15 seconds periodically, followed by centrifugation at maximum speed for 10 min [20].

### Western blotting

Immunoblotting was performed as described previously [22,23]. Primary antibodies were as follows: anti-BDNF rabbit polyclonal (Cat. # OSB00017W, 1:1000)(Thermo Fisher Scientific), anti-GSK-3β rabbit polyclonal (Cat. # AF1590, 1:1000)(R&D Systems, Minneapolis, MN), anti-BCL-2 rabbit polyclonal (Cat. # 3498S, 1:1000), anti-phospho-AKT (Ser473) rabbit polyclonal (Cat. # 9271S, 1:1000), anti-STAT3 rabbit polyclonal (Cat. # 9139, 1:1000), anti-ELAVL1 rabbit polyclonal (Cat. # 12582S, 1:1000), anti-Caspase-3 rabbit polyclonal (Cat. #9662S, 1:1000), anti-phospho-H2A.X (Ser139) rabbit polyclonal (Cat. # 2577S1:1000), anti-GADPH rabbit polyclonal (Cat. # 5174S, 1:1000), anti-cleaved Caspase 3 (9664S, 1:1000) rabbit polyclonal, anti-cleaved Caspase 7 rabbit polyclonal (8438S, 1:1000) (Cell Signaling, Danvers, MA), anti-COL3A1 rabbit polyclonal (Cat. # sc-514601, 1:100), anti-β-catenin rabbit polyclonal (Cat. # sc-7963, 1:200) (Santa Cruz, Dallas, TX), and anti-β-Actin mouse monoclonal antibody (Cat. # A5441, 1:5000) (Sigma, St. Louis, MO). All blots were scanned using an Odyssey Infrared Imaging System (LI-COR Biosciences, Lincoln, Nebraska). Band density was determined using LI-COR Image Studio Software.

### Measurement of B-type natriuretic peptide (BNP) and BDNF in serum and/or cytoplasmic/nuclear fractions

Serum levels of BNP were determined using a B-type Natriuretic Peptide EIA kit (Cat. # RAB0386, Sigma, St. Louis, MO). BDNF levels in the serum and cytoplasmic/nuclear fractions were quantitated using a rat BDNF ELISA kit (Cat. # EK0308, ScienCell Research Laboratories, Carlsbad, CA).

### Cell proliferation assay

Cell viability was assessed using the Vybrant® MTT Cell Proliferation Assay Kit (Thermo Fisher Scientific).

### MicroRNA microarray

MicroRNA microarrays were performed by LC Sciences (Houston, TX) using Microarray Version 21 (MRA-1003) [23]. Data were analyzed by subtracting the background and then normalizing the signals using a LOWESS (Locally-Weighted Regression) filter. The miRNA transcript was considered as reliably detectable only if the signal intensity was greater than 3 times the background standard deviation, the spot coefficient of variation was < 0.5, and at least 50% of the repeated probe signals were above the detection level.

### Statistical analysis

All data were expressed as mean ± SEM. We used one-way analysis of variance (ANOVA) followed by Tukey post-hoc analysis for comparison of more than two means, and Student’s t-test to compare between two means, and considered *p*<0.05 to be statistically significant.

## Results

### Chronic colitis impairs cardiac function

**Fig 1:**
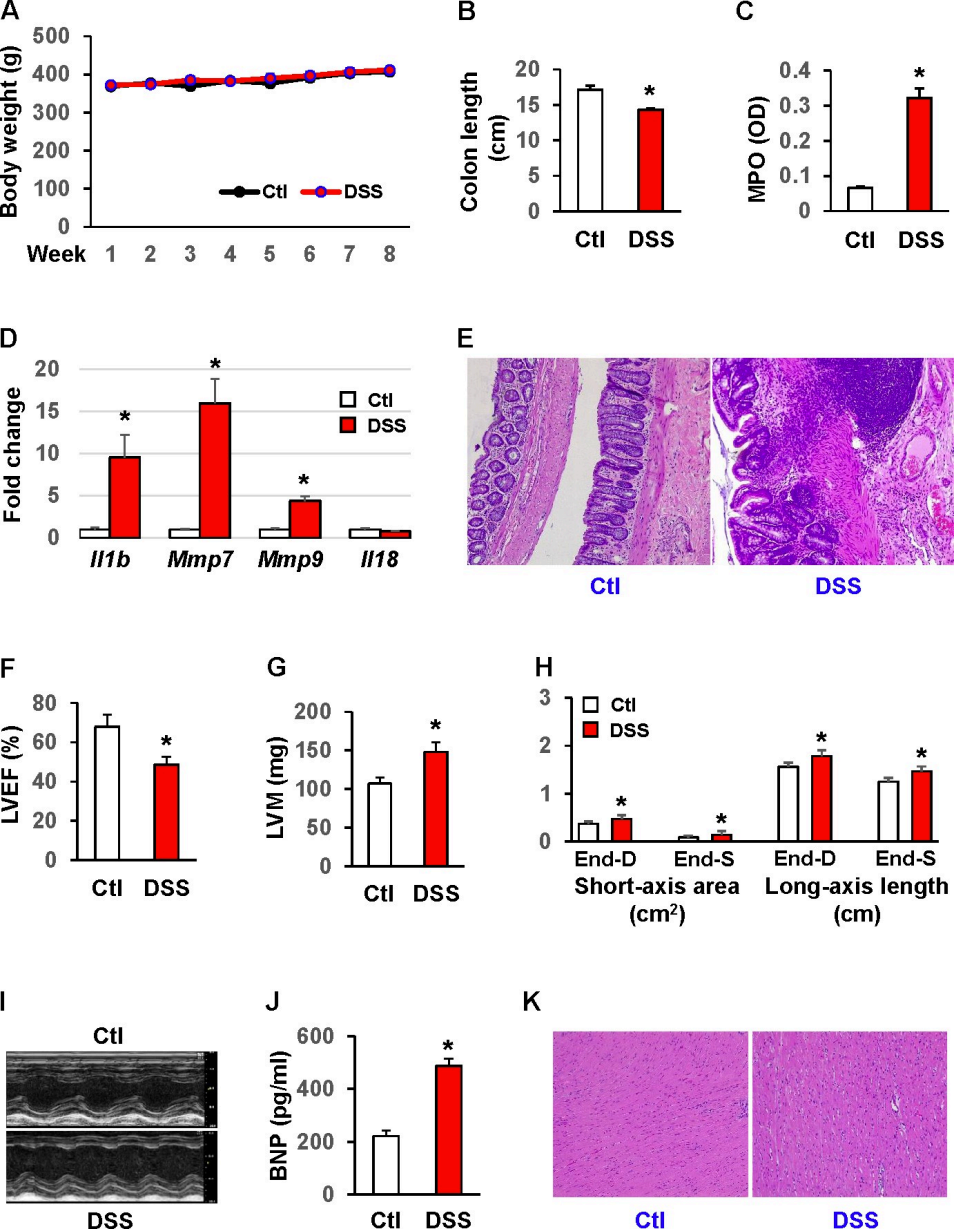
Chronic colitis impairs heart function in rats. Chronic colitis was induced with 3% DSS, which was applied for 4 cycles of 5 days each, with intermittent 9-day intervals of normal drinking water. (A) Chronic colitis had little effect on body weight. (B) Colon length was significantly shortened by DSS-induced colitis. MPO activities (C) and mRNA levels of *IL-1β*, *Mmp7*, and *Mmp9* but not *IL-18* were markedly elevated in the colonic mucosa of rats with chronic colitis (D). Messenger RNA (mRNA) levels were quantitated by RT-qPCR. (E) H&E staining of the colon. Echocardiography showed that chronic colitis significantly reduced LVEF (F) and increased LV mass (G) and size (H). LV sizes were presented as short-axis area (cm^2^) and long-axis length (cm). End-D, end-diastole; End-S, end-systole. (I) Representative M-mode images from rats with or without chronic colitis. (J) Chronic colitis also significantly elevated serum levels of BNP (pg/ml), indicative of heart failure. n = 8. * *p*<0.05 vs. control (Ctl). (K) Histological analysis of the rat heart by H&E staining.

To provide direct evidence that chronic colitis induces molecular remodeling in the adult heart, we first evaluated potential functional changes on the hearts of animals experiencing experimental colitis. Rats were exposed to 4 episodes of inflammation using 3% DSS to mimic the chronic, vacillating disease activity in IBD, a colitis model that has been very well characterized [24–26]. Three percent DSS caused no mortality and had no significant effect on the body weight compared to controls (Fig 1A). To confirm the colonic activity of DSS, the colon length was measured, which was significantly shortened after the exposure compared to controls (17.1±0.57 vs 14.3±0.21, *p*<0.05) (Fig 1B). In the setting of colonic inflammation mediated by DSS, myeloperoxidase activity (5-fold, Fig 1C) and mRNA levels of *IL-1β* (9.5-fold) [27–29], *Mmp 7*(16-fold), and *Mmp 9* (4.6-fold), but not *Il18* (Fig 1D), were significantly increased (*p*<0.01). H&E staining revealed that DSS rats had significant alterations in the overall mucosal architecture (Fig 1E), including irregular crypts with variable diameters along the depth of single crypts or dilated crypts, bifurcation at the base of the crypt, and crypts herniated to the submucosa. Cryptitis, crypt abscesses, and erosions, i.e. the loss of surface epithelium, were observed in some areas. The DSS rats demonstrated a significant decline in LVEF (30%, Fig 1F) and accompanying increases in left ventricular mass (36%, Fig 1G) and size (Fig 1H) compared to controls calculated on M-mode images from transthoracic echocardiography (Fig 1I). These physiologic changes noted in chronic colitis were accompanied by an increase in serum levels of BNP (2.4-fold, Fig 1J), a biochemical marker for heart failure, which is strongly associated with LV hypertrophy [30]. H&E staining of the heart sections showed cell swelling, irregular nuclear pattern, and enlarged spaces between muscle fibers in DSS rats (Fig 1K).

### MiRNA profiling identifies cardiac miRNAs significantly altered by DSS colitis

In an effort to identify cardiac miRNA expression changes mediated by chronic colitis, miRNA microarrays were performed on the cardiac tissue of DSS and control groups (S1 Fig). A hierarchical cluster analysis was performed for all miRNAs with a signal intensity >32. A t-test was performed comparing control groups to DSS rats (S2 Fig). Sixty-eight miRNAs with high signal (>500) had significant changes in expression levels (*p*<0.05). Thirty miRNAs were significantly increased (Table 1) and 38 were decreased (S1 Table) (*p*<0.05). Fifty-two miRNAs with low signal (<500) also had significant changes, among which 26 miRNAs including miR-155 were upregulated and 26 miRNAs downregulated (*p*<0.05). The data was deposited in the GEO Repository (Accession No. GSE158991. URL: https://www.ncbi.nlm.nih.gov/geo/query/acc.cgi?acc=GSE158991).

**Table 1:** Cardiac miRNAs significantly upregulated by DSS colitis.

miRNA Name	p-value	Control Mean	DSS Mean	Log2(G2/G1)
rno-let-7d-3p	4.53E-03	467	1,822	1.96
rno-miR-466c-5p	7.36E-03	213	545	1.36
rno-miR-485-3p	7.61E-03	790	2,420	1.62
rno-miR-1b	7.72E-03	7,455	64,823	3.12
rno-miR-32-3p	1.04E-02	575	1,779	1.63
rno-miR-181b-5p	1.10E-02	144	560	1.96
rno-miR-486	1.33E-02	813	3,746	2.20
rno-miR-483-5p	1.38E-02	564	2,250	2.00
rno-miR-466d	1.39E-02	325	778	1.26
rno-let-7i-5p	1.50E-02	1,813	9,201	2.34
rno-miR-568	1.60E-02	1,014	2,743	1.44
rno-let-7d-5p	1.70E-02	1,707	16,929	3.31
rno-miR-466b-3p	1.97E-02	3,202	10,198	1.67
rno-miR-466c-3p	2.06E-02	2,907	8,467	1.54
rno-miR-466b-5p	2.36E-02	693	1,594	1.20
rno-miR-98-5p	2.41E-02	52	619	3.58
rno-miR-146a-5p	2.43E-02	1,226	3,078	1.33
rno-let-7f-5p	2.54E-02	1,677	18,931	3.50
rno-miR-466b-2-3p	2.54E-02	1,864	5,137	1.46
rno-miR-1-3p	2.56E-02	3,916	40,178	3.36
rno-miR-210-5p	2.64E-02	862	2,219	1.36
rno-miR-206-3p	2.96E-02	241	546	1.18
rno-miR-423-5p	2.97E-02	287	811	1.50
rno-let-7a-5p	3.05E-02	2,057	19,963	3.28
rno-let-7b-5p	3.84E-02	2,020	13,764	2.77
rno-let-7e-5p	3.84E-02	266	2,535	3.25
rno-miR-352	3.87E-02	236	3,055	3.69
rno-let-7c-5p	3.97E-02	2,521	18,395	2.87
rno-miR-1896	4.35E-02	1,262	4,821	1.93
rno-miR-196c-3p	4.43E-02	1,695	7,092	2.06

### MiRNAs putatively targeting the BDNF gene were predicted and confirmed by RT-qPCR

**Fig 2:**
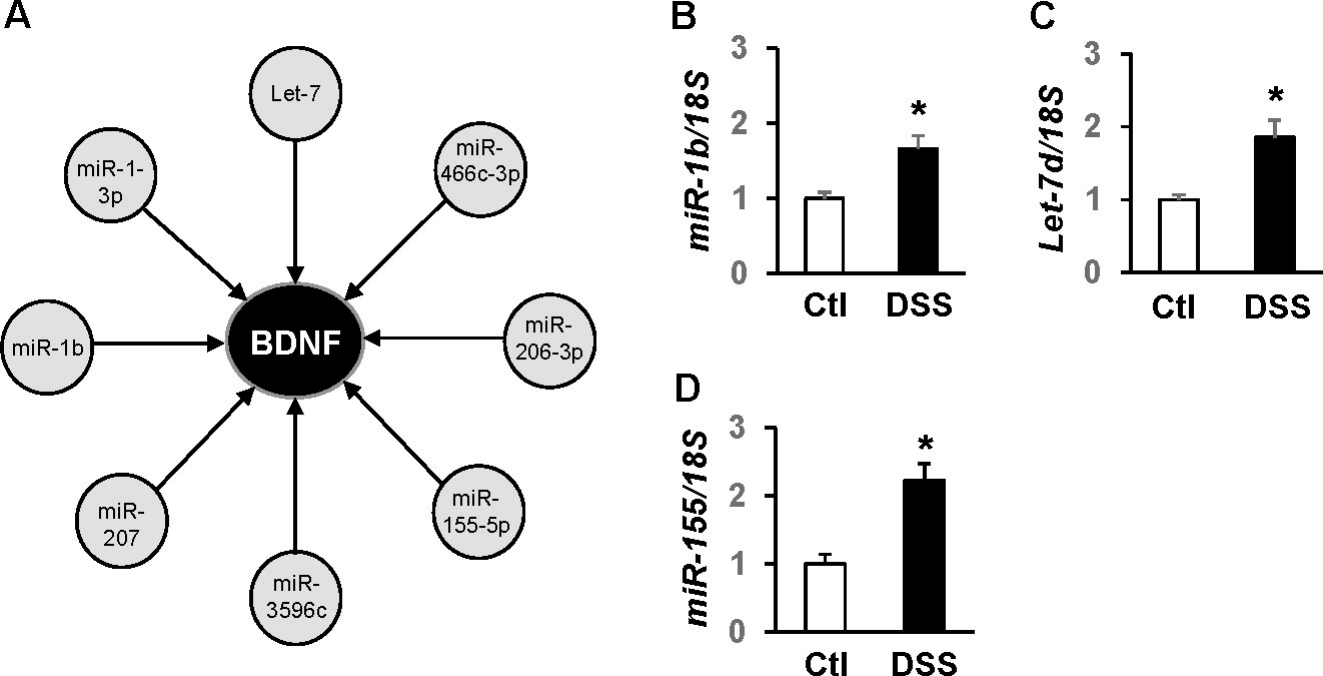
Multiple miRNAs targeting BDNF are elevated by colitis in the adult heart. Potential targets of altered miRNA were predicted using microRNA.org, miRDB, and miRWalk. (A) Eight miRNAs upregulated by DSS in miRNA microarrays were predicted as potential regulators of BDNF. RT-qPCR confirmed that miR-1b (B), Let-7d (C) and miR-155-5p (D) were significantly elevated by DSS colitis. No significant changes were found for miR-466c-3p, 1-3p, 206-3p, 3596c, and 207 (data not presented). n = 8. *p<0.01.

Using microRNA.org, miRDB, and miRWalk, target genes of all significantly altered miRNAs by DSS colitis were predicted. Interestingly, a total of 8 miRNAs increased by colitis in microRNA microarrays were predicted as regulators of BDNF (Fig 2A). RT-qPCR was subsequently performed, which confirmed that DSS colitis augmented levels of three of the miRNAs in cardiac tissue: miR-1b, Let-7d, and miR-155-5p (1.7-, 1.9-, and 2.3-fold, respectively. *p*<0.05) (Fig 2B–2D). No significant changes were found for miR-466c-3p, 206-3p, 3596c, 207, and 1-3p (*p*>0.05).

### MiR-1b, Let-7d, and miR-155 suppress BDNF in H9c2 cells

**Fig 3:**
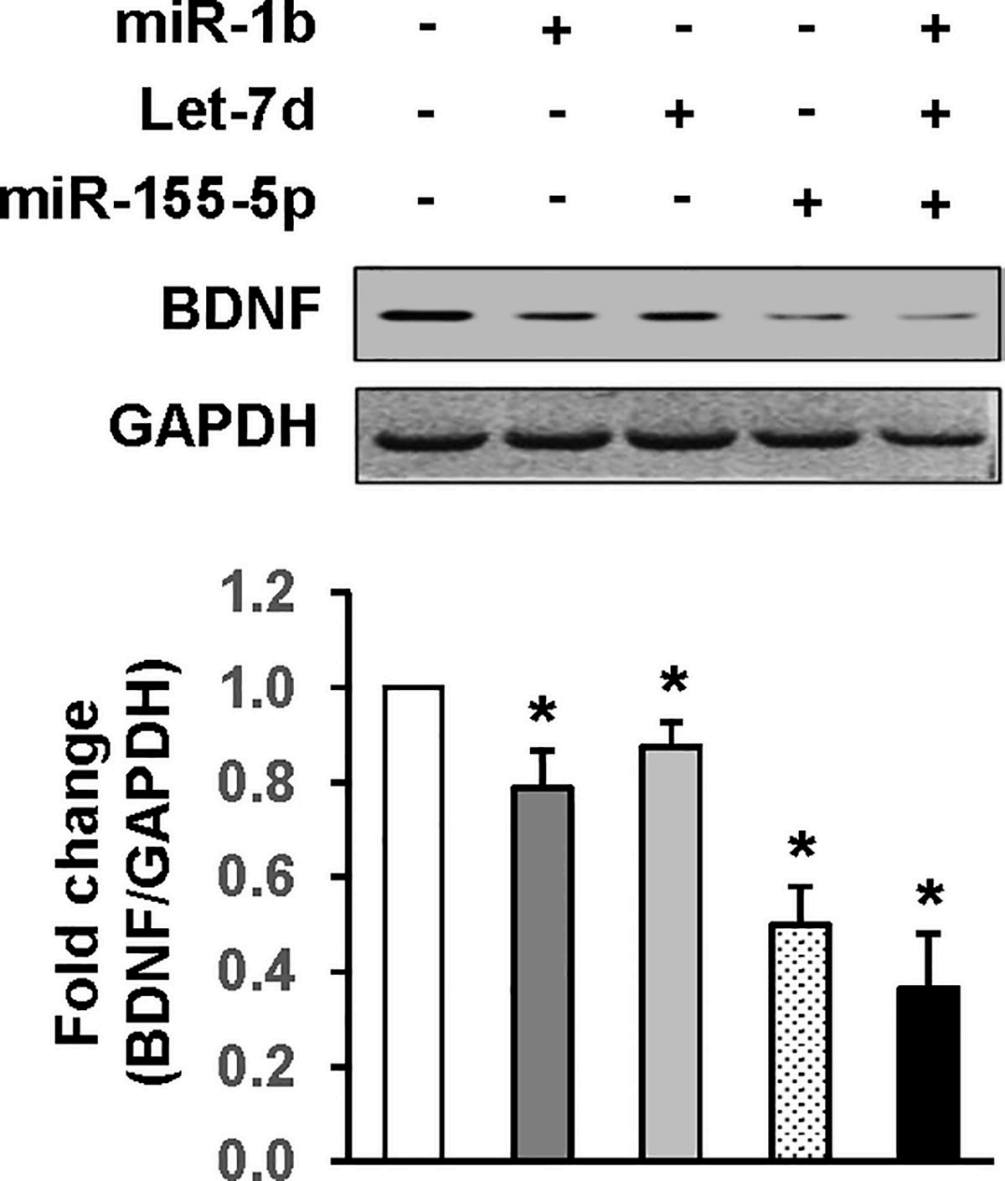
miR-1b, Let-7d, and miR-155 suppress BDNF in H9c2 cells. Mimics of miR-1b, Let-7d, and miR-155 or their combination were overexpressed in H9c2 cells by transient transfection. Top panel: Western blots showing miR-1b, Let-7d, and miR-155-5p, either alone or in combination, suppressed BDNF protein expression. Bar graph: Relative band density normalized to the housekeeping gene GAPDH. n = 3 independent experiment. * *p*<0.05 vs. control.

To confirm BDNF suppression by miR-1b, Let-7d, and miR-155, H9c2 cardiac myofibrobast cells were transfected with the miRNA of interest (Fig 3). All three miRNAs significantly downregulated BDNF protein expression compared to miRNA negative control (22%, 15%, and 43%, respectively, vs. control. *p*<0.05), with miR-155 demonstrating the most significant effect (*p*<0.05 vs. miR-1b and Let-7d). This effect was augmented by concurrent transfection of cells with all three miRNAs (*p*<0.01 vs. miR-1b and Let-7d).

### Loss of BDNF in rat models of experimental colitis

**Fig 4:**
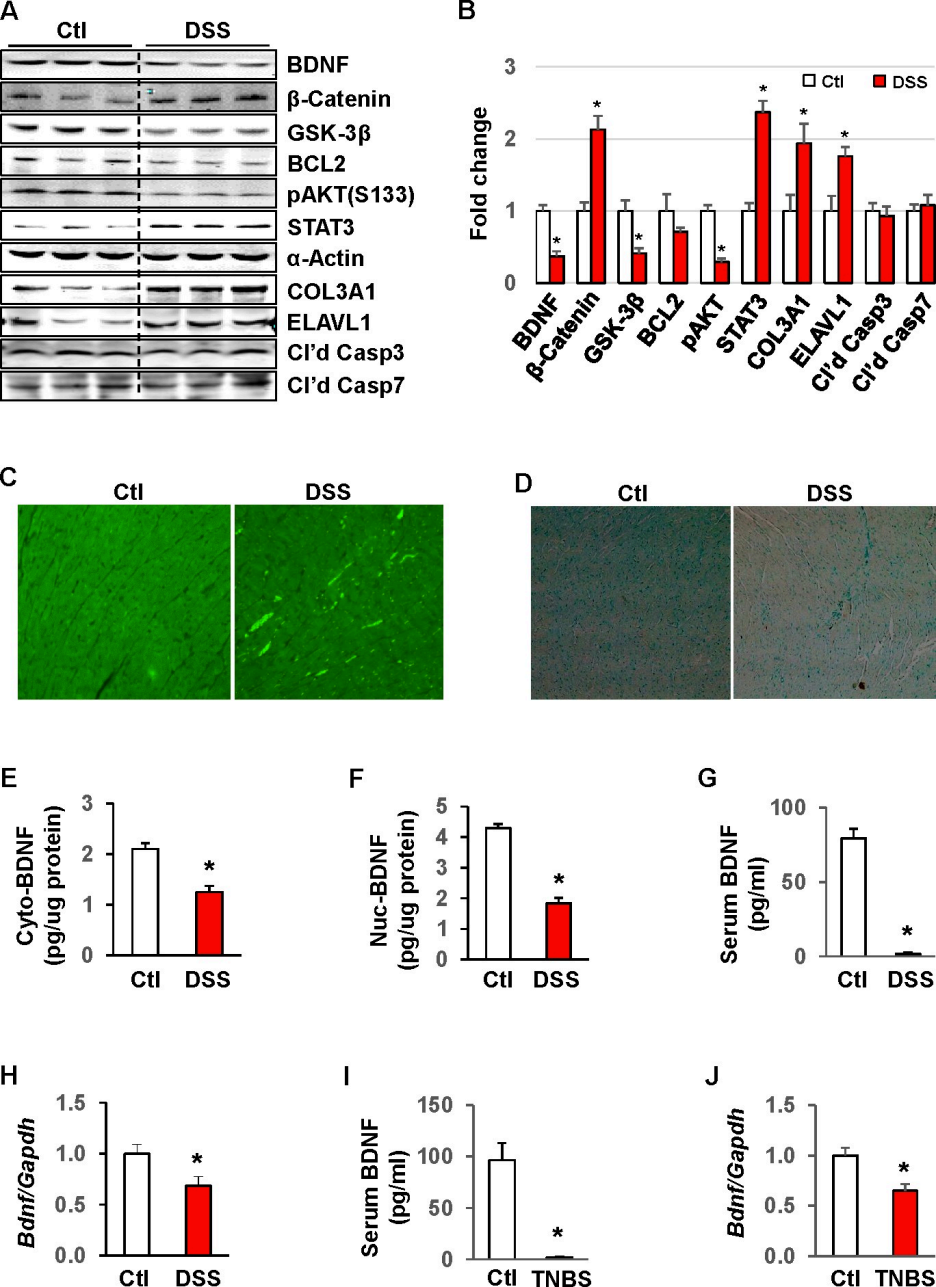
Chronic colitis induces marked changes in protein expression in the adult heart and blood. (A) Expression levels of proteins indicated in the tissue lysates of myocardium were significantly altered by DSS-induced chronic colitis. Western blot was performed to detect protein levels. Alpha-actin served as control. Cl’d Casp, cleaved Caspase. (B) Arbitrary optical density units of the targeting proteins were normalized *vs*. α-Actin and presented as fold change. (C) Immunostaining of COL3A1 in heart sections showing COL3A1 accumulation in DSS rats. (D) TUNEL staining of rat heart sections. Both cytoplasmic (E) and nuclear (F) BDNF protein levels in the heart were significantly decreased by DSS. BDNF protein levels in the extracts were assessed by enzyme-linked immunosorbent assay (ELISA). (G) BDNF levels in the serum was also dramatically downregulated by chronic colitis. (H) DSS colitis suppressed *BDNF* mRNA in the adult heart, which was quantitated by RT-qPCR with Gapdh as endogenous control. n = 8. *p<0.05 vs. control (Ctl). BDNF protein levels in the serum (I) and mRNA expression in the heart (J) were also significantly downregulated by TNBS-induced colitis. n = 6. *p<0.05 vs. control (Ctl).

In order to explore if changes in cardiac miRNA are associated with BDNF reduction, we examined the alterations in expression of BDNF[17–19], as well as notable cardiac proteins in DSS rats compared to age-matched controls: β-catenin, whose re-activation in cardiomyocytes triggers hypertrophic response via activation of mitogen-activated protein kinases [31,32]; BCL2, which has both pro and anti-apoptotic activity in heart failure [33]; phosphorylated AKT (pAKT), which is associated with short term increases in contractile function and efficient oxygen utilization in the myocardium [34]; STAT3, which is a downstream regulator of gp130 signaling associated with compensatory hypertrophy during periods of cardiac pressure overload [35]; COL3A1, which is a marker of cardiac fibrosis; ELAV-like protein 1 (ELAVL1), which is an important mediator of pyroptosis in inflammatory mediated heart failure [36]; and finally cleaved Caspases 3 and 7, whose enzymatic activity is necessary to complete apoptotic cell death [37]. In chronic colitis, BDNF protein levels in the myocardium were significantly reduced, accompanied by decreases in GSK-3β, BCL2, pAKT(S133) and increases in β-catenin, STAT3, COL3A1, and ELAVL1 compared to that in age-matched healthy controls (Fig 4A and 4B). Accumulation of COL3A1 in the DSS rat heart was confirmed by immunofluorescence, suggestive of cardiac fibrosis (Fig 4C). TUNEL staining showed very few positive apoptotic cells in both control and DSS rats (Fig 4D). Subcellular fractionation followed by enzyme-linked immunosorbent assay revealed that both cytoplasmic and nuclear BDNF levels in the adult heart were significantly attenuated by DSS (43% and 58%, respectively, *p*<0.05) (Fig 4E and 4F). This was accompanied by a significant reduction in circulating BDNF (91%, *p*<0.01) (Fig 4G). DSS induced colitis also suppressed BDNF mRNA expression in the adult heart (30%, *p*<0.05) (Fig 4H). In order to validate the changes induced by DSS, we also assessed BDNF expression in TNBS-induced acute colitis in adult rats, another well characterized rodent model of colitis [24,38]. BDNF protein levels in the serum and mRNA expression in the heart were significantly reduced by TNBS-induced colitis compared to controls (95% and 33%, respectively, *p*<0.05) (Fig 4I and 4J), confirming that both acute and chronic inflammation in the colon suppresses BDNF levels in the heart.

### BDNF ameliorates H_2_O_2_-induced apoptosis in H9c2 rat cardiomyoblast cells

**Fig 5:**
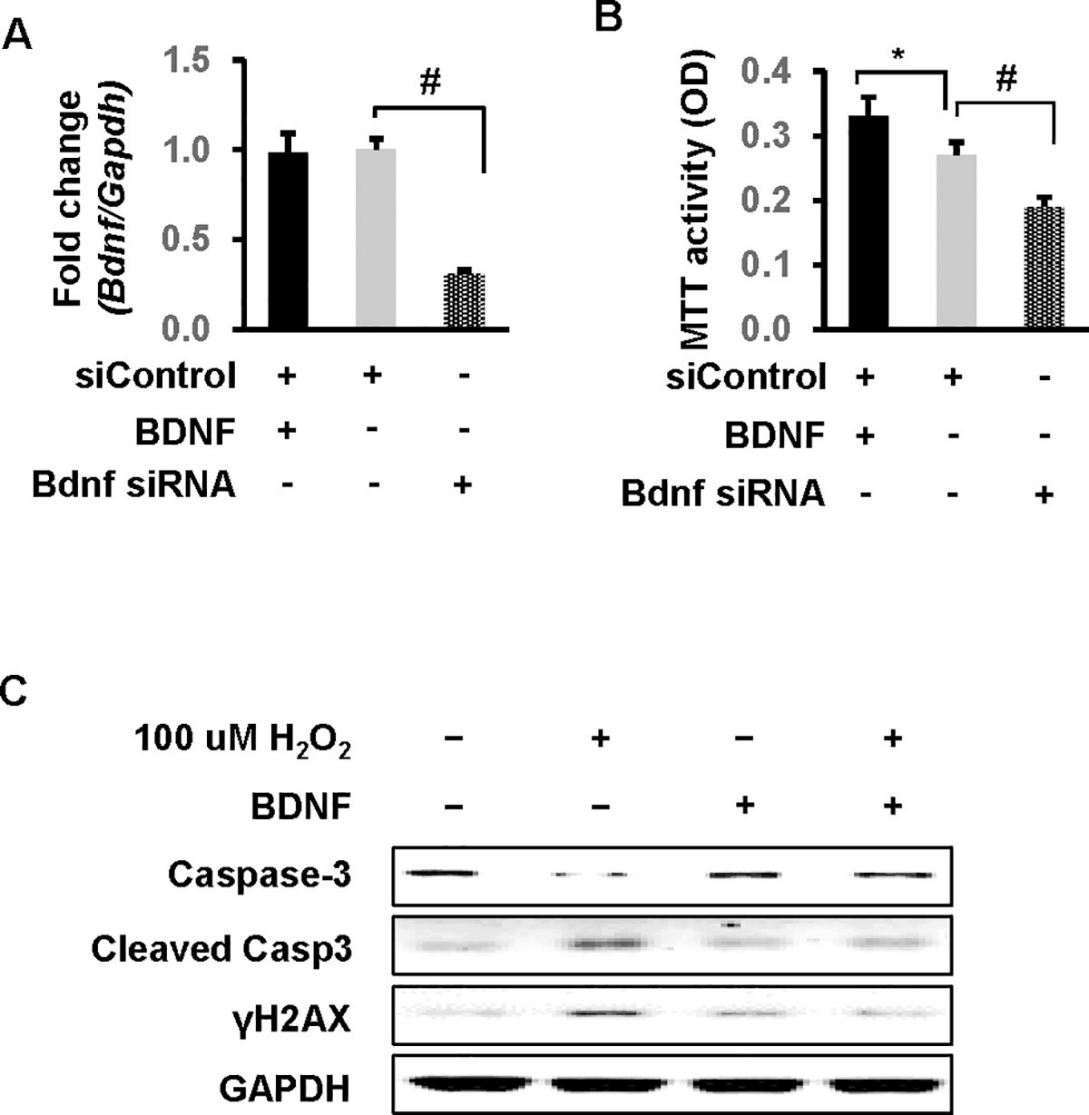
BDNF is critical to cell survival in H9c2 rat cardiomyoblast cells. (A) Cells were treated with recombinant BDNF protein or transfected with rat BDNF-specific siRNA. RT-qPCR was performed to confirm BDNF knockdown by siRNA. (B) Cell viability was significantly increased by recombinant BDNF protein and markedly suppressed by BDNF knockdown. Cell viability was evaluated by MTT assay. n = 3 independent experiments. * *p*<0.05, # *p*<0.01. (C) Forced expression of BDNF ameliorated H_2_O_2_-induced increases of cleaved caspase 3 and γH2AX, known markers of apoptosis. BDNF was overexpressed by transient transfection with empty vector as negative control. Proteins were detected by Western blot. GAPDH served as a loading control.

To help establish further the functional importance of BDNF in the adult heart [39], we investigated if BDNF impairs apoptosis in cardiomyocytes. While recombinant BDNF protein had no effect on BDNF mRNA levels, the rat BDNF-specific siRNA was successfully transfected into H9c2 rat cardiomyoblast cells with a significant reduction in BDNF mRNA (70%, *p*<0.01) (Fig 5A). Cell proliferation quantified by MTT colorimetric assay was significantly increased in the cells treated with recombinant BDNF protein compared to control cells (31%, *p*<0.05). A significant reduction in cell proliferation was noted in the BDNF knockdown group (35%, *p*<0.01 vs. siControl group) (Fig 5B). Apoptosis in H9c2 cells was induced through the administration of H_2_O_2_, manifested through cleavage of Caspase-3 and elevation of phosphorylated histone H2AX (γH2AX) (Fig 5C, left plate); the changes were mitigated by BDNF overexpression (Fig 5C, right plate). These findings demonstrate a protective role of BDNF in cardiomyoblasts.

### IL-1β increases miR-155 and decreases BDNF levels in the adult rat heart

**Fig 6:**
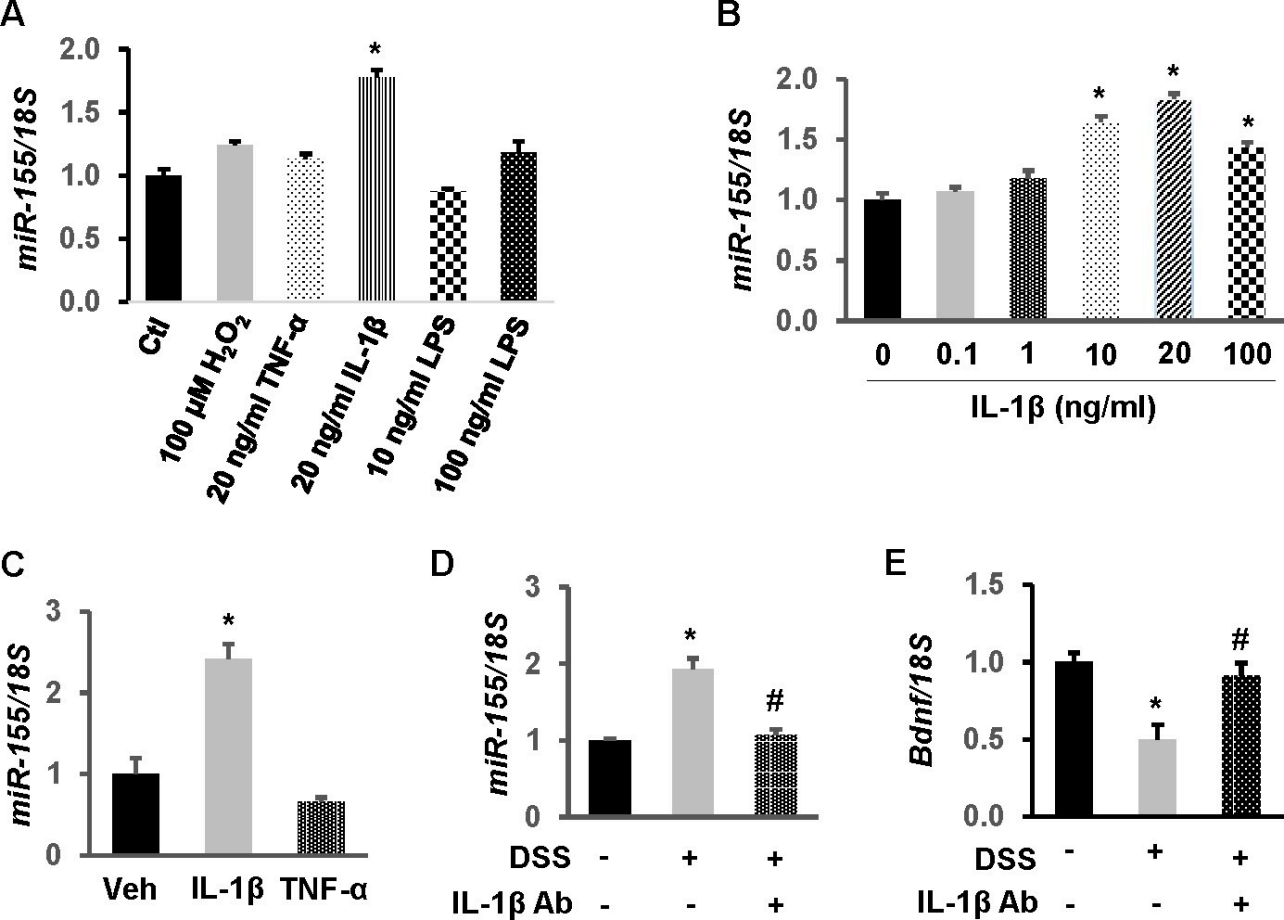
IL-1β mediates miR-155 upregulation by colitis in cardiomyocytes. (A) IL-1β upregulated miR-155 in H9c2 cells. Cells were treated with H_2_O_2_, TNF-α, IL-1β or LPS for 24 hours. miR-155 levels were quantitated by TaqMan-based RT-qPCR with 18S ribosomal RNA as endogenous control. (B) IL-1β elevated miR-155 in a dose-dependently manner. H9c2 cells were treated for 24 hours. n = 3 independent experiments. *p<0.01 vs. control (Ctl) or concentration zero. (C) Recombinant IL-1β, but not TNF-α, significantly elevated miR-155 in the adult rat heart *in vivo*. n = 8. *p*<0.01 vs. vehicle (Veh)-treated rats. (D) IL-1β neutralizing antibody ameliorated DSS-induced upregulation of miR-155 in the adult heart. (E) IL-1β blockade negated DSS-induced downregulation of BDNF in the adult heart. n = 6. *p<0.01 vs control, #*p*<0.05 vs. DSS alone.

To determine how systemic inflammation influences miR-155 expression in cardiac myocytes, independent experiments were performed to identify mediators of chronic inflammation augmenting myocyte expression of miR-155. First, H9c2 cells were treated with inflammatory mediators H_2_O_2_, TNF-α, IL-1β, or low and high dose LPS. Only IL-1β upregulated miR-155 levels significantly (1.7-fold, *p*<0.01) (Fig 6A). Elevated levels of miR-155 responded in a dose dependent manner up to concentrations of 20 ng/mL (10 and 20 ng/mL *p*<0.01 vs. concentration zero) (Fig 6B). To verify that IL-1β stimulates miR-155 in rat myocardium, we treated adult rats with recombinant rat IL-1β or TNF-α and evaluated miR-155 levels in cardiac tissue by RT-qPCR. These *in vivo* studies demonstrated that IL-1β, but not TNF-α, augments miR-155 in the adult rat hearts compared to vehicle treated controls (2.4-fold, *p*<0.01 vs. control and TNF-α) (Fig 6C). To further confirm that IL-1β alters miR-155 expression in the chronic colitis model, IL-1β neutralizing antibody was concurrently administered in DSS rats. IL-1β neutralizing antibody negated the DSS-induced upregulation of miR-155 (Fig 6D) in the adult rat heart (*p*<0.01 vs. vehicle-treated DSS rats). More importantly, downregulation of BDNF by DSS was concurrently abrogated by administration of IL-1β neutralizing antibody (Fig 6E).

## Discussion

Population-based studies found that IBD is associated with increased risk of hospitalization for heart failure [3,40], developing ischemic stroke [41], and myocardial infarction [17,42]. More recent studies have identified extracardiac sources of pro-inflammatory cytokines which can induce cardiac pump dysfunction [43]. Circulating inflammatory mediators, such as TNF-α, have been noted to correlate with disease severity in HF patients, and elevated levels are prognosticators of poor clinical outcomes [44]. In addition, patients presenting with HF with the highest quartile of circulating IL-1β had increased all-cause mortality at one year compared to their comorbidity matched cohorts [45]. The etiology of this chronic inflammatory state has not entirely been elucidated, although is thought to be mediated in part by increased release of endotoxins originating from the intestines [46]. In this study, we showed functional and anatomical changes of reduced LVEF corresponding with hypertrophic ventricular changes using trans-thoracic echocardiography in rats with chronic colitis, after demonstrating baseline echocardiographic results consistent with previously described rat controls [47]. These findings corresponded with biochemical evidence of heart failure with a 2.5 fold increase in BNP from control rats. This supports the population-based data that colitis can induce more severe phenotypes of HF and reinforces a connection between colonic integrity and extracolonic consequences in the heart. More importantly, we identified IL-1β as a major regulator of cardiac miR-155, which in turn suppressed heart BDNF. The significance of IL-1β in the chronic colitis model was reinforced when DSS-induced augmentation of miR-155 expression was shown to be reversed with IL-1β neutralizing antibody. These findings indicate that IL-1β is one of the missing links between IBD and HF. Blocking IL-1β might benefit patients with IBD by preventing cardiac remodeling that could lead to heart failure. Theoretically, IL-1β neutralization could also benefit patients with chronic autoinflammatory diseases such as rheumatoid arthritis [48], as well as type 2 diabetes, which is also considered as a chronic low-grade inflammatory disease [49].

We previously identified miR-155 as an important modulator of epithelial integrity in the setting of chronic colitis via its interference of colonic tight junctions by decreasing E-cadherin expression [23]. Let-7 has significantly altered expression in both Crohn’s disease and ulcerative colitis, albeit in contradictory patterns [50]. In cardiovascular disease, in addition to the before mentioned identification of miR-155 as a potential biomarker for non-ischemic cardiomyopathy, Let-7 has well established alterations of expression patterns in various cardiovascular diseases, notably upregulated in heart failure and ventricular hypertrophy [51]. miRNA profiling of the mouse adult hearts revealed a significant proportion of miR-1 of known miRNA reads [52]. When exposed to a cardiomyocyte-specific deletion of a gene required for miRNA production, LV malformations leading to dilated cardiomyopathy with subsequent LV dysfunction (manifested by diminished fractional shortening) were noted [53,54]. We utilized microarray analysis to identify the rat miRNAs significantly modulated by DSS colitis, and isolated the mRNAs predicted to target BDNF. Three miRNAs, miRNA-155, Let-7, and miR-1, were significantly increased in DSS colitis, and their common target, BDNF, was decreased significantly in cells transfected with the miRNA of interest. Prominent was miR-155, which was upregulated by IL-1β both *in vitro* and *in vivo*.

BDNF represents an emerging target for cardiovascular research due to its diverse effects of heart centric homeostasis [13] with alterations in expression in this growth factor implicated in a wide variety of cardiovascular diseases. We demonstrated that in response to chronic colitis, both serum and heart BDNF levels were significantly downregulated, which could adversely influence heart function. Importantly these changes were accompanied by alterations in protein expression of mediators which collectively are associated with molecular and biochemical modifications leading to hypertrophy (Fig 1K), fibrosis (Fig 4C), and the promotion of HF. BDNF knockdowns demonstrated significant reductions in cell proliferation in cardiomyoblast cells (Fig 5). Conversely, forced expression of BDNF reduced H_2_O_2_-induced apoptosis. In total, these findings support BDNF’s role as a mediator of chronic colitis-induced cardiac dysfunction.

The DSS model of induced colitis is considered a good preclinical model that exhibits many phenotypic features of relevance to human IBD [55–58]. DSS mice have increased anxiety-like behavior [59] and decreased electroretinography amplitudes, a measurement of retinal function [60], suggestive of two common extraintestinal manifestations. Our findings indicate that the DSS rat model could be a useful tool for mechanistic elucidation of cardiovascular diseases in IBD. However, rodent DSS models have their limitations as no animal models can fully recapitulate human IBD phenotypes and symptoms. Another limitation in this study is that the exact origins of miR-155 measured in the hearts remain to be determined. While our results suggest that IL-1β elevates miR-155 in cardiomyocytes, this event might also occur in the colon. High levels of miR-155 of gut origin could be transported to the heart by exosomes since miR-155 was found to be the most highly expressed miRNA in the blood samples of ulcerative colitis patients [11]. miR-155 is also highly elevated in the colon mucosa and feces collected from Crohn’s disease and ulcerative colitis patients [61,62].

While this study helps shed light on cardiac remodeling in IBD, we are aware that the human heart differs from the murine heart. Future studies could include procuring human heart tissue from deceased IBD patients and deceased control subjects without IBD, and comprehensive characterization of their molecular differences. Our findings suggest that IL-1β plays a major role in mediating cardiac remodeling through IL-1β→miR-155→BDNF signaling axis. IL-1β neutralization might be further tested for its ability to ameliorate HF in different preclinical models of colitis before consideration of proceeding to human clinical trials. While BDNF supplementation and miR-155 antagonists should be explored for their therapeutic potential in mitigating IBD-induced cardiac remodeling/HF, we cannot rule out other factors that may contribute to cardiac remodeling in IBD. For example, studies demonstrated a significant reduction in the diversity of the stool microbiome of individuals with IBD. The microbiota of patients with IBD is characterized by depletions in butyrate-producing bacteria with anti-inflammatory effects and an expansion in pathogenic bacteria [63]. While inflammation can be well controlled by drug therapy or surgery, gut microbial dysbiosis persists and may have a life-long impact on IBD patients. Thus, future studies should also investigate how colitis-induced microbiota dysbiosis influences the cardiac system.

In summary, the present study demonstrated that chronic colitis induced by DSS suppresses BDNF, a cardiac modulating neurotrophin with wide-ranging effects on normal cardiovascular development. This change was accompanied by the altered expression of proteins associated with biochemical and structural alterations associated with HF. We confirmed that BDNF ameliorated H_2_O_2_-induced apoptosis in H9c2 cardiomyoblast cells. We noted that three miRNAs, particularly miR-155, were predicted to be and then confirmed as regulators diminishing BDNF expression. Finally, we mechanistically connect chronic colitis-induced inflammation and miR-155-mediated changes in cardiomyocytes through IL-1β. These findings, in summation, provide an explanation to the previously noted relationship between HF and IBD. This supports the idea that chronic inflammation, and the modulation of its molecular byproducts, continues to represent a compelling area of therapeutic investigation particularly in patients with comorbid cardiovascular disease and colitis.

## Supporting information

S1 Fig Chip images of all 6 samples (n = 3). (PDF)

S2 Fig Clustering graphs of miRNA microarrays. (A) Clustering graph of all miRNAs with signal intensity >32 (all detectable miRNAs). Altered miRNAs were also clustered based on the p values. (B) *P*<0.1. (C)*P*<0.05. (D)*P*<0.01.
(PDF)

S1 Table Cardiac miRNAs significantly decreased by DSS colitis. (PDF)
