# Supplementary figures and images for: Chronic colitis upregulates microRNAs suppressing brain-derived neurotrophic factor in the adult heart
Source: PLoS One. 2021 Sep 20;16(9):e0257280. doi: 10.1371/journal.pone.0257280 (PMC8452076; doi:10.1371/journal.pone.0257280)

Control

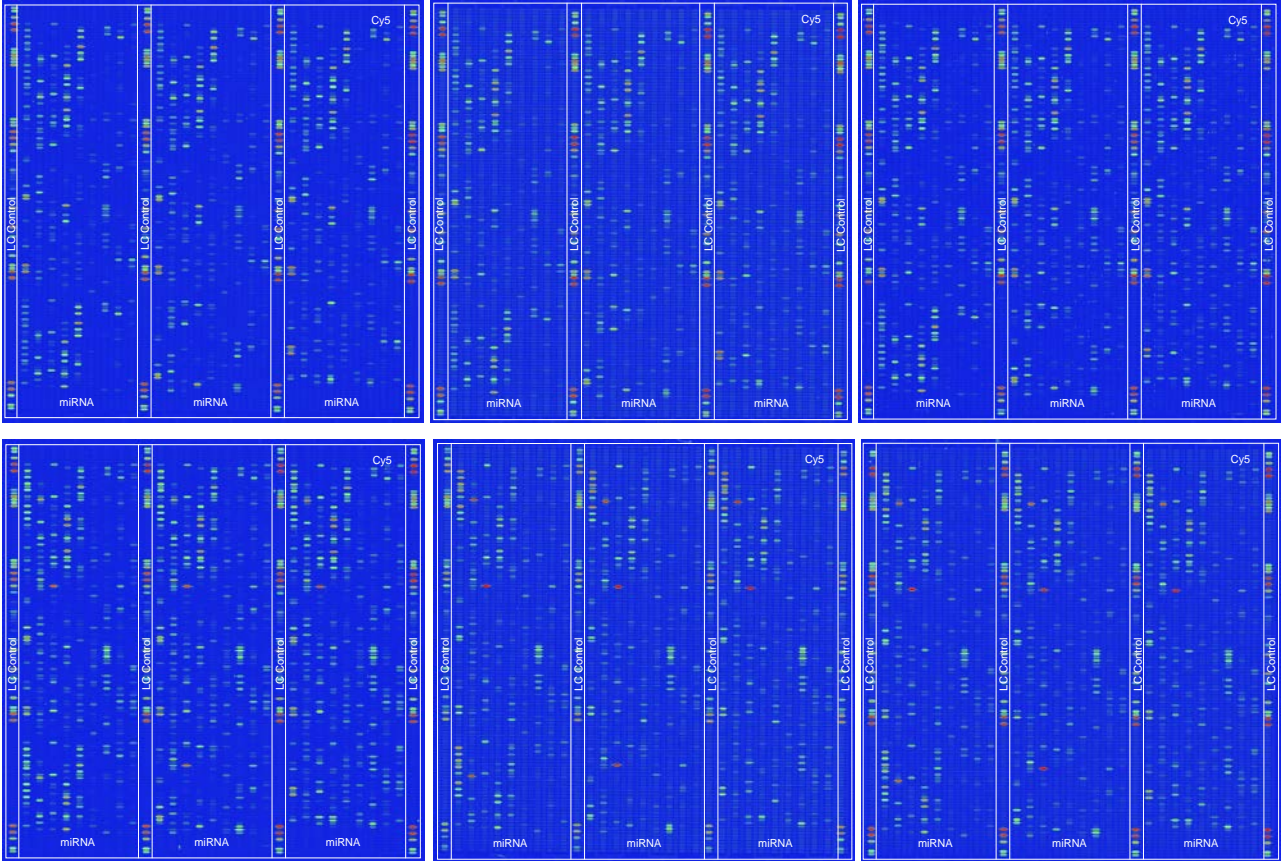

DSS

Fig S1. Chip images of all 6 samples (n=3).

Supplement: S1 Fig — (PDF) [file pone.0257280.s001.pdf]
