# Supplementary material for: Chronic colitis upregulates microRNAs suppressing brain-derived neurotrophic factor in the adult heart
Source: PLoS One. 2021 Sep 20;16(9):e0257280. doi: 10.1371/journal.pone.0257280 (PMC8452076; doi:10.1371/journal.pone.0257280)
Supplement: S2 Fig — (A) Clustering graph of all miRNAs with signal intensity >32 (all detectable miRNAs). Altered miRNAs were also clustered based on the p values. (B) P<0.1. (C)P<0.05. (D)P<0.01. (PDF) [file pone.0257280.s002.pdf]

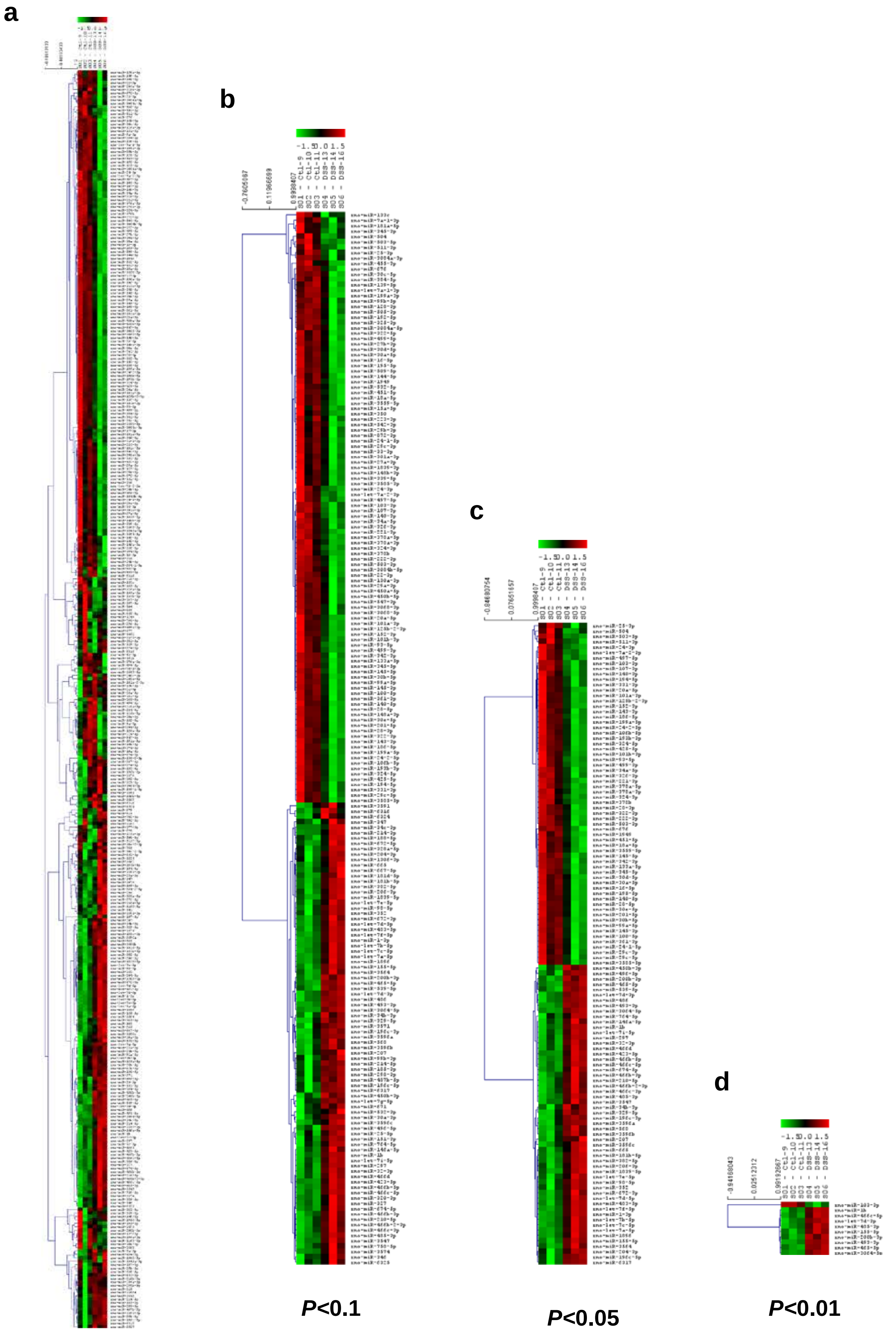

Fig S2. Clustering graphs of miRNA microarrays. (A) Clustering graph of all miRNAs with signal intensity >32 (all detectable miRNAs). Altered miRNAs were also clustered based on the p values. (B) *P*<0.1. (C) *P*<0.05. (D) *P*<0.01.
