# Supplementary material for: Chronic colitis upregulates microRNAs suppressing brain-derived neurotrophic factor in the adult heart
Source: PLoS One. 2021 Sep 20;16(9):e0257280. doi: 10.1371/journal.pone.0257280 (PMC8452076; doi:10.1371/journal.pone.0257280)
Supplement: S1 Table — (PDF) [file pone.0257280.s003.pdf]

Table S1. Cardiac miRNAs significantly decreased by DSS colitis

miRNA Name	p-value	Control Mean	DSS Mean	Log2(G2/G1)
rno-miR-103-3p	8.48E-03	1,491	842	-0.82
rno-miR-145-5p	1.06E-02	9,562	4,926	-0.96
rno-miR-107-3p	1.22E-02	1,467	845	-0.79
rno-miR-378a-5p	1.91E-02	421	112	-1.91
rno-miR-99a-5p	1.93E-02	5,001	877	-2.51
rno-miR-30b-5p	1.95E-02	8,765	2,148	-2.03
rno-miR-425-5p	2.03E-02	476	72	-2.72
rno-miR-186-5p	2.05E-02	993	294	-1.75
rno-miR-322-3p	2.14E-02	485	116	-2.06
rno-miR-152-3p	2.30E-02	1,223	405	-1.59
rno-miR-101b-3p	2.38E-02	302	47	-2.67
rno-miR-221-3p	2.53E-02	552	282	-0.97
rno-miR-199a-5p	2.65E-02	693	140	-2.30
rno-miR-140-3p	2.68E-02	448	171	-1.39
rno-miR-100-5p	2.71E-02	3,293	725	-2.18
rno-miR-451-5p	2.87E-02	3,701	473	-2.97
rno-miR-133a-5p	2.95E-02	1,214	340	-1.84
rno-miR-24-2-5p	2.98E-02	628	113	-2.48
rno-miR-106b-5p	2.98E-02	364	53	-2.79
rno-miR-101a-3p	3.02E-02	722	63	-3.52
rno-miR-378b	3.02E-02	2,019	1,210	-0.74
rno-miR-195-5p	3.05E-02	4,780	1,582	-1.60
rno-miR-28-5p	3.10E-02	516	177	-1.54
rno-miR-30a-5p	3.14E-02	8,318	2,872	-1.53
rno-miR-30e-5p	3.15E-02	6,530	1,186	-2.46
rno-miR-143-3p	3.23E-02	5,588	3,899	-0.52
rno-miR-20a-5p	3.27E-02	722	203	-1.83
rno-miR-16-5p	3.49E-02	4,862	1,459	-1.74
rno-miR-497-5p	3.54E-02	433	150	-1.53
rno-miR-30d-5p	3.74E-02	8,182	3,721	-1.14
rno-miR-378a-3p	3.89E-02	2,406	1,489	-0.69
rno-miR-93-5p	4.11E-02	375	123	-1.61
rno-miR-342-3p	4.13E-02	602	312	-0.95
rno-miR-194-5p	4.23E-02	342	76	-2.18
rno-miR-25-3p	4.35E-02	1,004	590	-0.77
rno-miR-24-3p	4.36E-02	9,023	6,297	-0.52
rno-miR-145-3p	4.79E-02	833	160	-2.38
rno-miR-29c-3p	4.82E-02	5,855	689	-3.09
